# EEG-Based Emotion Recognition Using a 2D CNN with Different Kernels

**DOI:** 10.3390/bioengineering9060231

**Published:** 2022-05-26

**Authors:** Yuqi Wang, Lijun Zhang, Pan Xia, Peng Wang, Xianxiang Chen, Lidong Du, Zhen Fang, Mingyan Du

**Affiliations:** 1 Institute of Microelectronics of Chinese Academy of Sciences, Beijing 100029, China; wangyuqi182@mails.ucas.edu.cn (Y.W.); zhanglijun@ime.ac.cn (L.Z.); 2 University of Chinese Academy of Sciences, Beijing 100049, China; xiapan17@mails.ucas.edu.cn (P.X.); wangpeng01@aircas.ac.cn (P.W.); chenxx@aircas.ac.cn (X.C.); lddu@mail.ie.ac.cn (L.D.); 3 Aerospace Information Research Institute, Chinese Academy of Sciences (AIRCAS), Beijing 100190, China; 4 Personalized Management of Chronic Respiratory Disease, Chinese Academy of Medical Sciences, Beijing 100190, China; 5 China Beijing Luhe Hospital, Capital Medical University, Beijing 101199, China

**Keywords:** emotion recognition, machine learning, convolutional neural network, electroencephalogram

## Abstract

Emotion recognition is receiving significant attention in research on health care and Human-Computer Interaction (HCI). Due to the high correlation with emotion and the capability to affect deceptive external expressions such as voices and faces, Electroencephalogram (EEG) based emotion recognition methods have been globally accepted and widely applied. Recently, great improvements have been made in the development of machine learning for EEG-based emotion detection. However, there are still some major disadvantages in previous studies. Firstly, traditional machine learning methods require extracting features manually which is time-consuming and rely heavily on human experts. Secondly, to improve the model accuracies, many researchers used user-dependent models that lack generalization and universality. Moreover, there is still room for improvement in the recognition accuracies in most studies. Therefore, to overcome these shortcomings, an EEG-based novel deep neural network is proposed for emotion classification in this article. The proposed 2D CNN uses two convolutional kernels of different sizes to extract emotion-related features along both the time direction and the spatial direction. To verify the feasibility of the proposed model, the pubic emotion dataset DEAP is used in experiments. The results show accuracies of up to 99.99% and 99.98 for arousal and valence binary classification, respectively, which are encouraging for research and applications in the emotion recognition field.

## 1. Introduction

### 1.1. Background

Emotions are mainly the individuals’ inner responses (such as attention, memorization, achieving goals, awareness of priority, knowledge motivation, communication with others, learning development, mood status, and effort motivation) [1,2] to whether or not the objective conditions meet their psychological expectations which could also reflect their attitudes and perceptions [3]. Preliminary researchers in neuroscience, psychology, and cognitive science have confirmed that emotions play a key role in rational decision making, interpersonal communication, learning, memory, physical, and even mental health [4]. Long-term negative emotions and depression can interfere with individuals’ normal behaviors and health [5]. Affective computing is a branch of artificial intelligence that relates to, arises from, or influences emotions and it has emerged as a significant field of study that aims to develop systems that can automatically recognize emotions [2]. In the past decade, there have been a variety of methods to recognize emotions, including those based on self-evaluation (such as the Self-Assessment Manikin (SAM)) [6], which reports behavioral responses (such as vocal intonations, facial expressions, and body postures), and physiological signals. However, due to the social nature of human beings, individuals usually do not desire to express their real emotional states, they habitually disguise their expressions and movements when a camera is on them [7]. Moreover, to ensure the monitored data is of high quality, users are required to maintain a stable posture and position in front of the camera, microphone, and other sensors. These limitations lead it to be challenging to apply in a practical sense. By contrast, individuals are not able to disguise or conceal their physiological responses easily, thus, physiological signals can truly reflect the emotional changes of users. Consequently, methods of recognition based on physiological signals have become mainstream [8]. Among these physiological signals used in emotion recognition, electrocardiogram (ECG), respiratory (RSP), body temperature, etc. have their disadvantages since the variability of these signals is usually subtle and the change rate is typically slower. By contrast, electroencephalography (EEG) signals have the advantage of a high real-time differential and of being non-fakeable. In addition, emotion is essentially regulated by the central nervous system. Therefore, using EEG signals to recognize emotion is usually more accurate and objective than using other peripheral physiological signals [9,10,11,12,13]. EEG-based emotion recognition has attracted an increasing number of scholars and has been proved to be an effective method of emotion recognition in a multitude of research [14].

### 1.2. Related Work

#### 1.2.1. Emotion Model

**Figure 1:**
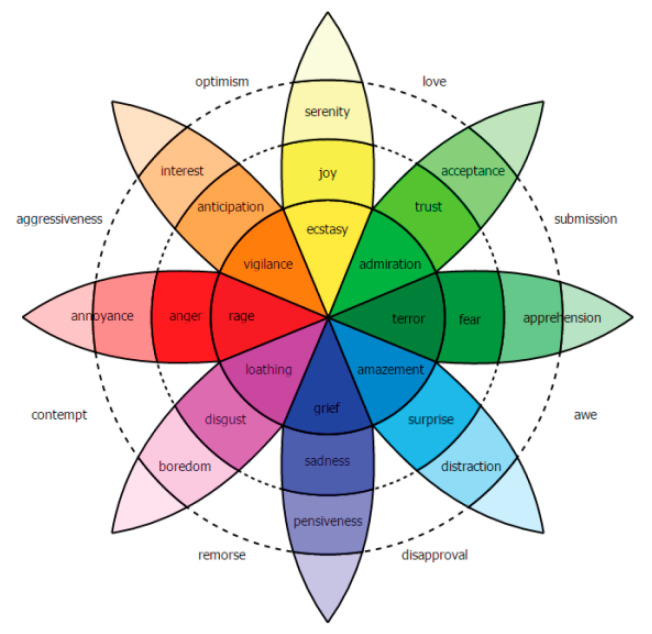
Plutchik emotion wheel.

Since people have different ways to express their emotions, judging their emotions is a challenging task. There are two main models of emotion. Some researchers hold the view that emotions are composed of several basic discrete emotions. For example, Ekman believes that emotions consist of happiness, sadness, fear, and anger. Under different cultural backgrounds and social environments, these basic emotions form more complex emotions by a combination in different forms [15]. By contrast, other researchers believe that emotional states are consecutive and indissociable. Plutchik proposed the famous emotion wheel model. As Figure 1 shows, the middle circle indicates the basic emotions. The outer circle and the inner circle respectively represent the undersaturation and the oversaturation of basic emotion [16].

**Figure 2:**
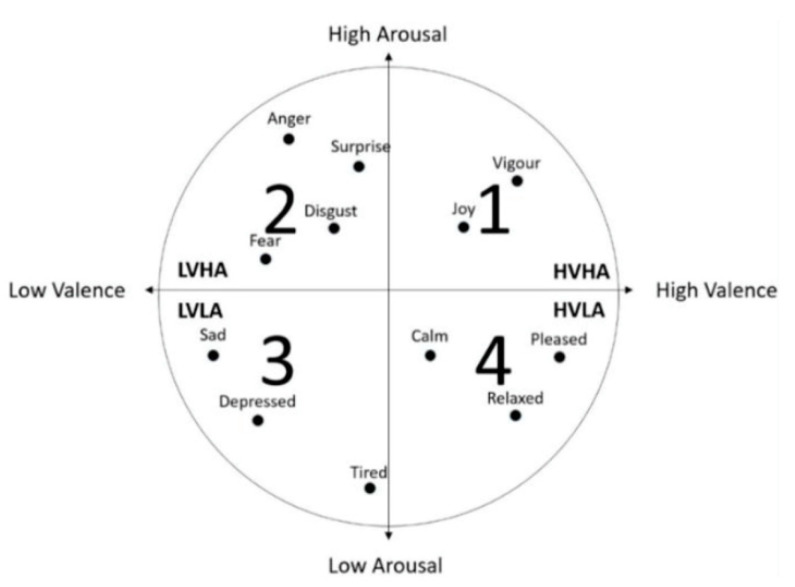
The Arousal-Valence model.

The most widely accepted emotion model is the two-dimensional model ‘Arousal-Valence’, proposed in 1980 by Russell. Figure 2 illustrates the Arousal-Valence model, the x-axis represents the Valence Dimension and the y-axis represents the Arousal Dimension. Different emotions can be located in this model. The emotion model used in the most popular public emotion dataset ‘DEAP’ is the extended version of ‘Arousal-Valence’.

#### 1.2.2. EEG-Based Emotion Recognition 

Most existing approaches are based on machine learning techniques for EEG emotion recognition [17]. For the classifiers in emotion recognition, the traditional machine learning algorithms such as support vector machine (SVM) and k-nearest neighbor (KNN) are frequently used and achieve good results. Kroupi designed a linear discriminant analysis model as the classification and used the power spectral density as a feature to recognize emotion [18]. Bahari used a k-Nearest to classify the emotion and got the accuracy of 64.56%, 58.05%, and 67.42% for three classes of arousal, valence, and liking [19]. Zheng proposed selecting 12 channel electrode features in SVM, which provided 86.65% on average [20].

However, the methods that use traditional machine learning algorithms have required the extraction of the emotion-related features from the origin EEG fragment. The extraction is time-consuming and the process uncertain. Moreover, the emotion recognition accuracies of these methods could be improved. Therefore, deep learning-based methods in emotion recognition have become increasingly popular. Tripathi used a DNN as the classifier to obtain better results as the accuracy of valence and arousal is 81.4 and 73.4%, respectively [21]. Zhang used the sparse autoencoder (SAE) and logistic regression to predict the emotion status. The recognition accuracy has improved to 81.21% for valence and 81.26% for arousal [22]. Nevertheless, the accuracy of emotion recognition by using CNN or SAE is still not high. Alhagry proposed a long-short term (LSTM) model to address emotion recognition. They used the DEAP dataset to test the method and the accuracies were 85.45% and 85.65% for valence and arousal, respectively [23]. Salama recognized emotions by a 3D convolutional neural network(3D-CNN) model. They extracted multi-channel EEG signals into 3D data for the Spatio-temporal feature extraction. The recognition accuracies on the DEAP dataset were improved to 87.44% and 88.49% for valence and arousal, respectively [24]. Song designed a dynamical graph convolutional neural network (DGCNN) which used graph relation to represent EEG and then use graph convolution network (GCN) to classify emotion. They tested the method on the DREAMER database and achieved the recognition accuracies of 86.23%, 84.54%, and 85.02% for valence, arousal, and dominance, respectively [25]. Zhong presented a regularized graph neural network (RGNN) to capture both local and global inter-channel relations and the accuracy is 85.30 on the SEED dataset [26]. Yin combined the graph CNN and LSTM. They took advantage of both methods to extract the graph domain features for emotion recognition and attained the average classification accuracy of 84.81% and 85.27% for valence and arousal [27]. Yang subtracted the Base Mean outcome from raw EEG data, then the processed data were converted to 2D EEG frames. They proposed a fusion model of CNN and LSTM and achieved high performance with a mean accuracy of 90.80% and 91.03% on valence and arousal classification tasks respectively [28]. Liu used a deep neural network and sparse autoencoder combined model to classify emotion [29]. Zhang tried many kinds of deep learning methods to classify emotions and got the best performance by using a CNN-LSTM model (accuarcy:94.17%) [30]. Donmez designed their own experiment and collected an emotion dataset, then used CNN to classify it and obtained an accuracy of 84.69% [31]. There are also some studies whose goals are not concerned with emotional recognization, but the methods they used for the classification of EEG signals using deep learning are worth learning from. For example, Abdani used Convolutional Neural Networks to test subjects to determine whether the task is new or routine [32], and Anwar used the AlexNet to classify motor imagery tasks [33].

### 1.3. The Contributions of This Study

There are still three main limitations, however, that need to be addressed in the studies in the field of emotion recognition to improve performance. The first one is the feature extraction problem. The shallow traditional machine learning models such as SVM and KNN used in emotion recognition require researchers to extract the emotional-related features manually as the input of their models. Some studies that used the deep learning model also extracted features manually to improve the classification performance. Manually extracting these features is time-consuming and the quality of these extracted features is also unstable given the involvement of human subjective consciousness and experience. Another main issue is that researchers used the user-dependent model to improve their model’s performance. The training and testing data are chosen from the same subject in the User-dependent model. Thus, the User-dependent emotion recognition model typically shows high accuracy. Nevertheless, the User-dependent model lacks generalization and universality. For every different subject, the User-dependent model requires training data of the specific individual to perform the tuning process, which means the model needs to be retrained every time the subject changes. The accuracies of most emotion recognition models are not high enough, thus there is still room for improvement. To address the above issues, we, therefore, propose a novel 2D-CNN model with two different sizes of convolution kernels that convolve along with the time and space directions, respectively. The proposed model does not need to manually extract the emotional-related features. The emotion recognition classification result could be directly derived from the raw EEG data by the proposed model, which realizes the end-to-end functionality. Furthermore, the proposed model is a User-independent model that is more applicable to new users because there is no need to create a new model for every single subject. The model just needs to be trained once for all the subjects and it could effectively monitor the emotion of a new user. Finally, the effectiveness of our model is examined on the DEAP dataset. The proposed model achieves the state-of-the-art accuracy of 99.97% on the valence and 99.93% on the arousal. 

The layout of the paper is as follows: In Section 1, important background on emotion reignition is described and also previous works are reviewed. Section 2 begins by introducing the domains of EEG signals and how they will be used in emotion recognition. It then introduces the DEAP dataset and the process of the dataset. The experiment setup and the entire process of our model are presented in detail at the end of Section 2. Section 3 explains the results achieved by the proposed method to demonstrate its effectiveness. Finally, the conclusion of this work and future work follows in Section 4.

## 2. Materials and Methods

### 2.1. EEG on Emotion

As the reflection of the central nervous system (CNS), asynchronous activity occurs in different locations of the brain during emotions [34]. Therefore, EEG can reveal significant information on emotions [35]. EEG is a waveform recording system that reads scalp electrical activity generated by the human brain over a period of time. It measures voltage fluctuations resulting from the ionic current flowing through the neurons of the brain. The EEG signal has a low amplitude which ranges from 10 uV to 100 uV [36]. The frequency range of EEG signals is typically 0.5–100 Hz [37]. According to various mental states and conditions, researchers divide EEG signals into five frequency sub-bands that are named the delta (1–4 Hz), theta (4–7 Hz), alpha (8–13 Hz), beta (13–30 Hz), and gamma (>30 Hz), respectively [38].

### 2.2. The Dataset and Process

**Figure 3:**
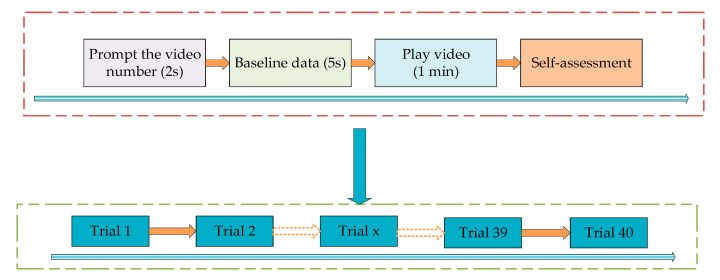
Experiment process.

The majority of the past studies about EEG—based emotion recognition in the last 10 years used the public open dataset to compare with other researchers and to demonstrate the advantages of their methods. The most used public open datasets are the MAHNOB-HCI, the DEAP, and the SEED. Among the articles conducted by public open dataset, most of them used the DEAP dataset [39]. To evaluate our proposed model, we adopted the most popular emotion dataset, DEAP, to conduct the experiments and verify the effectiveness. The DEAP (Database for Emotion Analysis using Physiological Signals) is a public open emotion dataset collected by researchers from the Queen Mary’s University of London, the University of Twente in the Netherlands, the University of Geneva in Switzerland, and the Swiss Federal Institute of Technology in Lausanne. They recorded the EEG and peripheral physiological signals of 32 healthy participants aged between 19 and 32 with an equal number of males and females. All 32 subjects were stimulated to produce certain emotions. Subjects were asked to relax for the first two minutes of the experiment. The first twominute recording is regarded as the baseline. Then subjects were asked to watch 40 music video excerpts of oneminute duration and these served as the sources of emotion elicitation. Figure 3 shows the process of the emotion elicitation experiment. 

**Figure 4:**
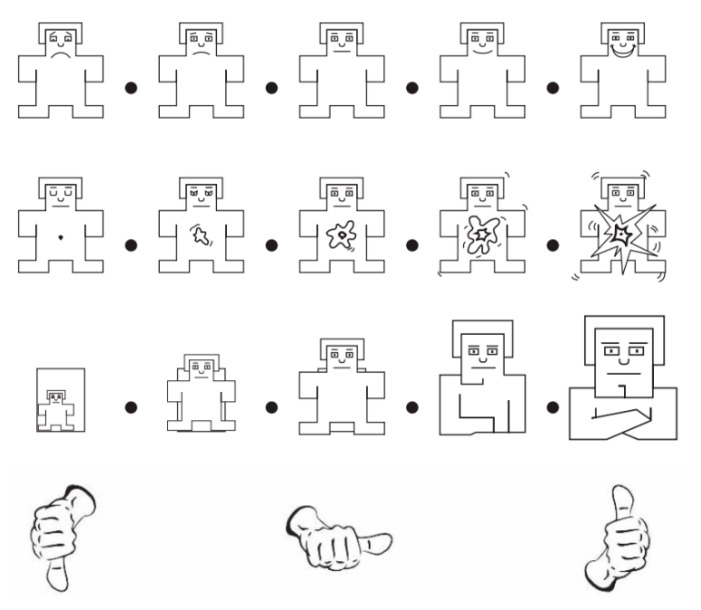
The self–assessment scales.

During this process, the EEG signals were recorded, at a sampling rate of 512 Hz, from 32 electrodes placed on the scalp according to the international 10–20 system [40]. At the same time, the peripheral physiological signals including skin temperature, blood volume pressure, electromyogram, and galvanic skin response were recorded from the other 8 channels. Every subject completed 40 trials corresponding to the 40 videos. As a result, there are 1280 (32 subjects × 40 trials) signal sequences in the DEAP dataset. For each trial, the first 3 s are used as a baseline because participants did not watch the videos during this time. Then they watched the one minute video. At the end of each trial, subjects were asked to perform a self–assessment to evaluate their emotional levels of arousal, valence, liking, and dominance in the range of 1 to 9. The self–assessment scales are a manikin designed by Morris, as shown in Figure 4 [41].

This scale represents from top to bottom, the levels of Valence, Arousal, Dominance, and Liking, respectively. Furthermore, DEAP provides a preprocessed version of the recorded EEG signals. In this study, we used the preprocessed version that the EEG signal is downsampled into 128 Hz. To reduce the noises and cut the EOG artifacts, the EEG signal was filtered by a band-pass filter with a frequency from 4 Hz to 45 Hz. The size of the physiological signal matrix for each subject is 40 × 40 × 8064, which corresponds to 40 trials × 40 channels × 8064 (128 Hz × 63 s) sampling points [40].

### 2.3. Experiment Setting

**Figure 5:**
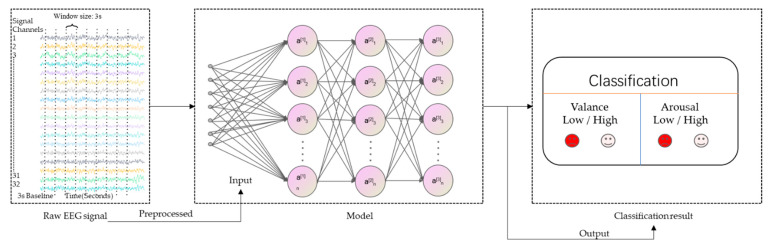
Process of emotion recognition using EEG.

Valence and Arousal are the most dimensional expression of basic emotions that researchers usually focus on. Therefore, in this study, we chose Valence and Arousal as the two scales for emotion recognition. Among the 40 channels of the DEAP dataset, we chose the first 32 channels that contain the EEG signal as the basis of emotion recognition. The labels of each data depend on the self-assessment rating values of Arousal and Valence states. We divide the rating values of 1-9 into two binary classification problems with a threshold of 5: If the self-assessment is more than 5, the label of this data is 1 (represents high valence/arousal), otherwise, the label of this data is 0 (represents to low valence/arousal). The process of recognizing emotions using EEG signals is shown in Figure 5.

The raw EEG signals from the DEAP original version were preprocessed to reduce the noise and cut the EOG artifacts. After this, the preprocessed version DEAP dataset was produced. Then the preprocessed raw EEG signals are extracted for the deep learning model. Finally, the emotion recognition classification results are obtained after the training and testing of the model.

### 2.4. Proposed Method

#### 2.4.1. Deep Learning Framework

To have the full benefit of the preprocessed raw EEG structure, we propose a two-dimensional (2D) Convolutional Neural Network (CNN) model in this study for emotion recognition. CNN is a class of deep neural networks widely used in a number of fields [42].

Compared with traditional machine learning methods, 2D CNN models have a better ability to detect shape-related features and complex edges. In a typical CNN network, there could be components named convolutional layers, pooling layers, dropout layers, and fully connected layers. Features from original EEG signals are concatenated into images and then sent to convolutional layers. After being convolved in convolutional layers, the data is further subsampled to smaller size images in pooling layers. During the process, network weights are learned iteratively through the backpropagation algorithm.

The input vector of the CNN structure is the two-dimensional feature, shown below:

(1) $$a=\left(\begin{array}{cccc} a_{11} & a_{12} & \ldots & a_{1n}\\ a_{21} & a_{22} & \ldots & a_{2n}\\ \ldots & \ldots & \ldots & \ldots \\ a_{m1} & a_{m2} & \ldots & a_{mn} \end{array}\right)$$

The shape of the input vector a is ***m*** × ***n***. Then the input vector a is convolved with ***Wk*** in the convolution layer, ***Wk*** is given by:

(2) $$c_{k}=\left(\begin{array}{c} c_{11}\\ c_{21}\\ \cdots \\ c_{i1} \end{array}\right)$$

In Formula (2), the length of $b_{k}$ is ***i*** which must be less than ***m*** in Formula (1). The feature map is finally obtained after the convolution, which is calculated as:

(3) $$f\left(\alpha \right)=f\left(c_{k}\times a+b_{k}\right)$$

After the convolutional layer, BatchNorm2d is used to normalize the data. This would keep the data size from being too large and prevent gradient explosion before the LeakyReLU layer. BatcgNorm2d is calculated as:

(4) $$y=\frac{x-mean\left(x\right)}{\sqrt{Var\left(x\right)}+\;\mathrm{eps}\;}*gamma+\;\mathrm{beta}$$

where x is the value of the input vector, mean(x) is the mean value and the $\sqrt{Var\left(x\right)}$ denotes the variance value. Eps is a small floating-point constant to ensure that the denominator is not zero. Gamma and beta are trainable vector parameters.

In Formula (3), f is the activation function, in this study, we use the leaky rectified linear unit (LeakyReLU).

LeakyReLU is defined in Formula (5):

(5) $$y_{\alpha }=\{\begin{array}{c} x_{\alpha }\;\;\;\;\;\mathrm{if}\;x_{\alpha }\geq 0\\ \frac{x_{\alpha }}{a_{\alpha }}\;\;\;\;\;\mathrm{if}\;x_{\alpha }<0 \end{array}$$

where α is defined in Formula (3) and $a_{i}$ is a fixed parameter in the range of 1 to +∞.

ReLU has more advantages in avoiding gradient disappearance than traditional neural network activation functions, such as *sigmoid* and *tanh*. LeakyReLU has the same advantage in avoiding gradient disappearance as ReLU. Moreover, during backpropagation, the gradient can also be calculated for the part whose input is less than zero by LeakyReLU (instead of having a value of zero as in ReLU). Thus, LeakyReLU can avoid the gradient direction sawtooth problem. The $b_{k}$ is the bias value, *k* is the filter, and the $\;c_{k}$ ∈ *Ri* × 1 is the weight matrix. The total number of filters in the convolutional layer is denoted by *n.*

**Figure 6:**
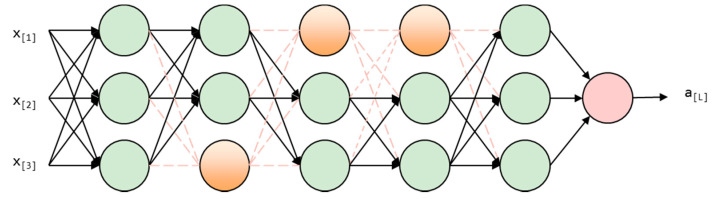
Dropout layer diagram.

A dropout of 0.25 is used in the Leaky Relu layer for reducing the network complexity and reducing over-fitting. Thus, enhancing the generalization of the model. As Figure 6 shows, some neural network units are temporarily discarded with a certain probability, in our model, the probability is 0.25.

**Figure 7:**
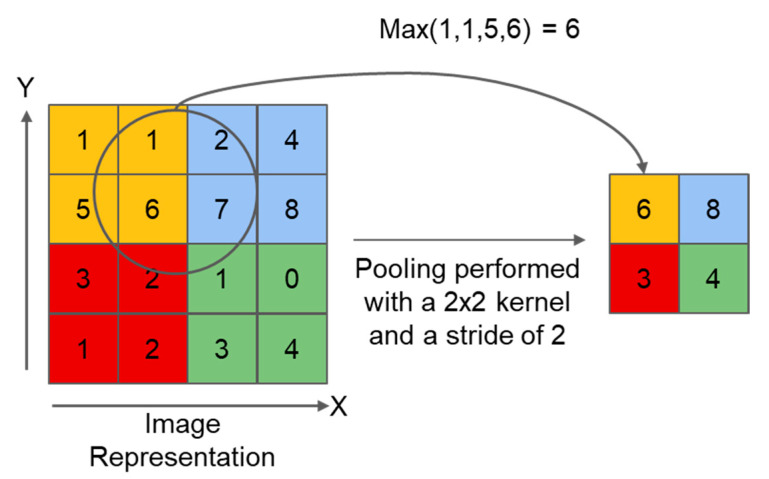
The process of Max pooling.

The feature map is then downsampled through the max-pooling layer. Figure 7 shows the principle of Max pooling.

The maximum value from the given feature map is extracted by this function. The fully connected layer is flattened after the last polling layer. Finally, since the task is a binary classification task, SoftMax is used in the output layer. Adam is used as the optimizer and binary CrossEntropy is applied to calculate the model loss because the labels of the arousal or valence classification are high value and low value. The Cross-Entropy in the binary-classification task is calculated as below:

(6) $$L=-\left[y^{*}log\hat{y}+\left(1-y\right)*log\left(1-\hat{y}\right)\right]$$

For the data with ***N*** samples, the calculation process is:

(7) $$L=-\sum_{n=1}^{N}{\hat{y}}_{i}logy_{i}+\left(1-{\hat{y}}_{i}\right)log\left(1-{\hat{y}}_{i}\right)$$

where ***N*** is the number of samples, y is the one-hot value, and $\hat{y}$ is the output.

#### 2.4.2. CNN Model

To better obtain the details of EEG signals associated with emotions, the 2D CNN is employed to feature extraction and classification in our study. Table 1 shows the major hyper-parameters and the related information, such as the value or the type of them of the proposed trained CNN model.

**Figure 8:**
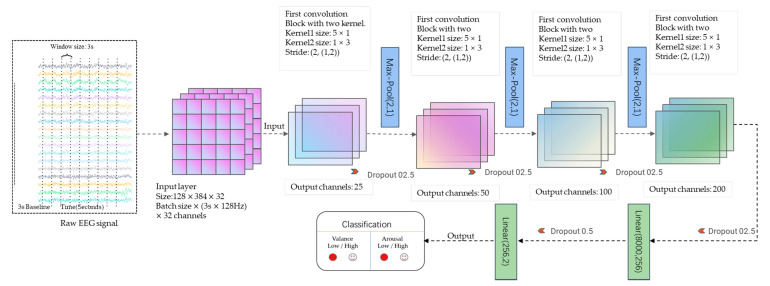
The architecture of the proposed network.

Our proposed CNN architecture is illustrated in Figure 8.

The model is designed using Python3.7. As Figure 8 and Table 1 show, the input size is width × height, where width is 32 (the number of electrode channels), and height equals 3× 128 = 384 ((window size: 3 s) × (sampling rate 128 Hz)). The batch size of the model is 128. So, the shape of the input data is (12,838,432). In the proposed model, Conv2 represents the dimensional (2D) convolutional layer, Pooling2D denotes 2D max pooling, BatchNorm2d is the 2D batch normalization, and Liner denotes the fully connected layer. Every convolution layer is followed by an activation layer, in this model we use the Leaky Relu as the activation function with the alpha = 0.3. The proposed model contains eight convolution layers, four batch normalizations, four drop-out layers with the probability of 0.25, three max-pooling layers, and two fully connected layers. All above layers collectively form the four convolution blocks. One convolution block has two convolution layers with two different convolution kernels to extract the emotion-relevant features. The two convolution kernels have distinct kernel sizes, in particular 5 × 1 and 1 × 3. The convolution kernel with size 5 × 1 convolves the data along the time direction and the other convolution kernel with size 1 × 3 convolves along the spatial direction. Every single convolution layer uses LeakyReLU as the activation function. Each convolution block has a normalization layer and a dropout layer (0.25). The first three convolution blocks are connected with Max pooling layers at the end of them. The last convolution block is followed by a fully connected layer and it is connected to the last output fully connected layer after a dropout layer with the probability of 0.5. The final output of this model is the classification results of the emotion recognition. Table 2 shows the shapes of the proposed model.

## 3. Results

We use the most popular public emotion dataset DEAP of the EEG signals to evaluate the proposed model. Seventy percent of the data of the DEAP dataset is randomly divided into the training set and the other thirty percent is the test set. The classification metric is the accuracy (ACC) which is the most commonly used evaluated guideline and represents the proportion of the sample that is classified correctly, given by:

(8) $$ACC=\frac{TP+TN}{TP+TN+FP+FN}\times 100\%\;$$

where ***TP***, ***TN***, ***FP***, and ***FN*** were denoted as the number of true positives, true negatives, false positives, and false negatives, respectively. 

**Figure 9:**
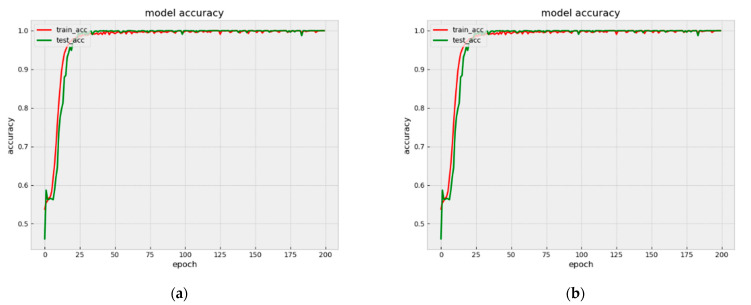
Model accuracy and loss curves (**a**) accuracy in Valence, (**b**) loss in Valence.

**Figure 10:**
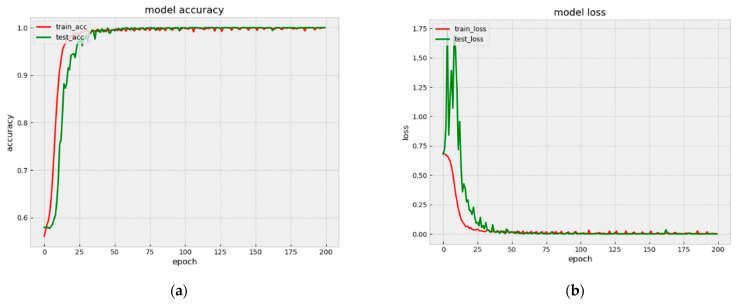
Model accuracy and loss curves (**a**) accuracy in Arousal, (**b**) loss in Arousal.

Our model achieves the accuracies of 99.99% and 99.98% on Arousal and Valence binary classification, respectively. The accuracy and loss classification of Valence and Arousal are shown in Figure 9 and Figure 10.

**Figure 11:**
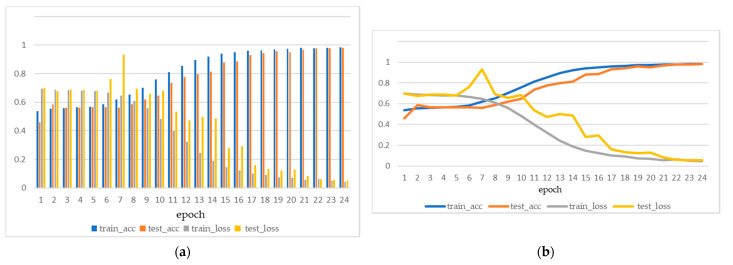
Model accuracy and loss curves during the first 25 epochs. (**a**) histogram, (**b**) line chart.

As can be seen from Figure 9 and Figure 10, for both Arousal and Valence classification, the proposed model converged quickly from epoch 0 to epoch 25. Figure 11 shows the Model accuracy and loss curves during the first 25 epochs. The model accuracies have already achieved 98.36% and 98.02% on Arousal and Valence after 25 epochs, respectively. Then from epoch 25 to epoch 50, the model accuracies improved slowly. Until the 50 epochs, the model accuracies are 99.56% and 99.68% on Arousal and Valence, respectively. Then the model is stabilizing during 50 to 200 epochs and finally achieves the high accuracies of 99.99% and 99.98% on Arousal and Valence, respectively.

**Figure 12:**
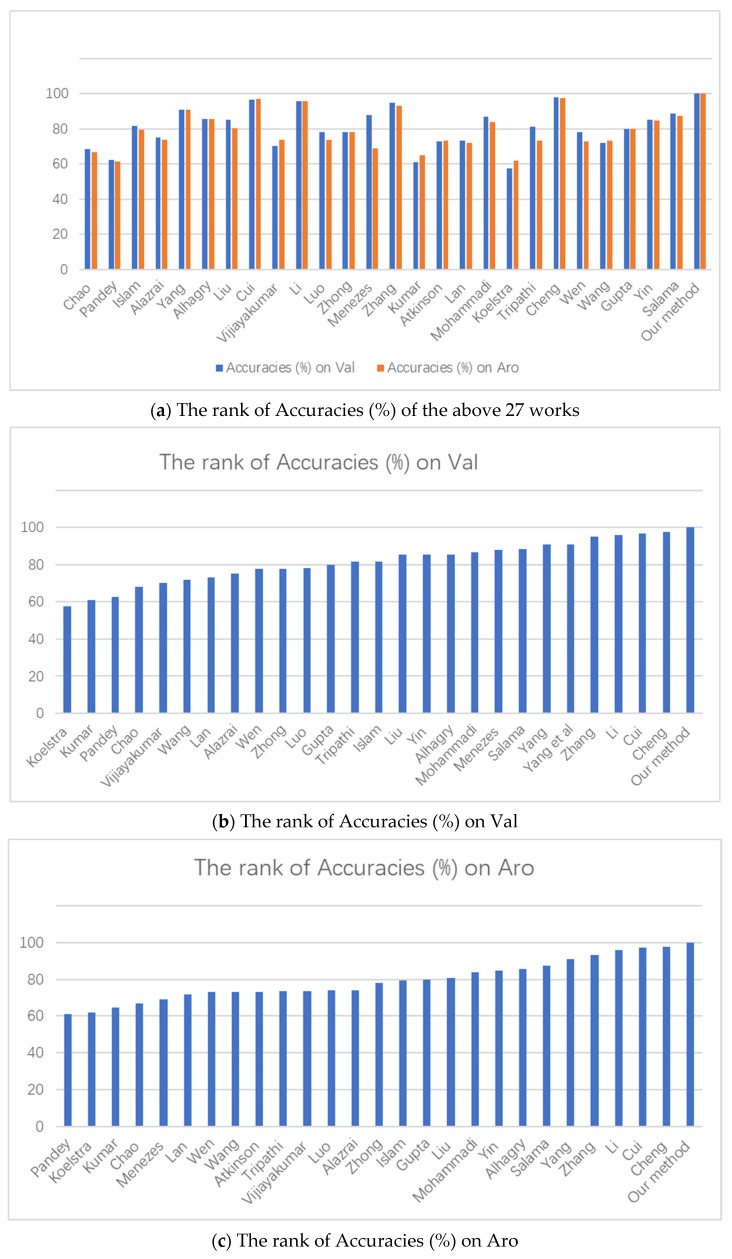
(**a**–**c**) Comparison in the form of histograms.

In Table 3, we compare the Arousal and Valence binary classification accuracies to other studies which used the DEAP dataset. From Table 3 and Figure 12, it can be concluded that among all the above methods of emotion recognition, our method has the best performance.

## 4. Conclusions

In this paper, we proposed the Convolutional Neural Network model with two different sizes of convolution kernels to recognize emotion from EEG signals. This model is user-independent and has high generalization and universality. It is more applicable to new users because there is no need to create a new model for every single subject. It also is an end-to-end model which is time-saving and stable. The effectiveness of our method is ascertained on the DEAP public dataset and the performance has been proved to be at the top of the area. Wearable devices have made great progress in people’s daily lives, and they have been widely used in the application of healthcare [63]. Measuring EEG signals with miniaturized wearable devices becomes possible. Future works will consider designing our own emotion experiments and transferring the model to other public emotion datasets such as the SEED dataset and evaluating the performance.

## Figures and Tables

**Table 1:** The value or types of the proposed model’s hyper-parameters.

Hyper-Parameter of the Proposed Model	Value/Type
Batch size	128
Learning rate	0.0001
Momentum	0.9
Dropout	0.25
Number of epochs	200
Pooling layer	Max pooling
Activation function Window size Optimizer Loss Function	LeakyReLU 3 S Adam Cross Entropy

**Table 2:** The shapes of the proposed model.

Number of Layers	Layer Type	Numbers of Input Channels/Output Channels
1	Input (shape:1, 384, 32)	
2	conv_1 (Conv2d)	1/25 (kernel size: 5 × 1)
3	droputout1 (Dropout=0.25)	1/25
4	conv_2 (Conv2d)	25/25 (kernel size: 1 × 3, stride = (1,2))
5	bn1 (BatchNorm2d)	25
6	pool1 (MaxPool2d (2,1))	25/25
7 8 9 10 11 12 13 14 15 16 17 18 19 20 21 22 23 24	conv_3 (Conv2d) droputout2 (Dropout = 0.25) conv_4 (Conv2d) bn2 (BatchNorm2d)pool2 (MaxPool2d (2,1)) conv_5 (Conv2d) droputout3 (Dropout = 0.25) conv_6 (Conv2d) bn3 (BatchNorm2d) pool3 (MaxPool2d (2,1)) conv_7 (Conv2d) droputout4 (Dropout = 0.25) conv_8 (Conv2d) bn4 (BatchNorm2d) flatten (Flatten layer) Linear1 (Linear) Droputout5 (Dropout = 0.5) Linear2 (Linear)	25/50 (kernel size: 5 × 1) 25/50 50/50 (kernel size: 1 × 3, stride = (1,2)) 50 50 50/100 (kernel size: 5 × 1) 50/100 100/100 (kernel size: 1 × 3, stride = (1,2)) 100 100 100/200 (kernel size: 5 × 1) 100/200 200/200 (kernel size: 1 × 3) 200 Shape: 128 × 8000 8000/256 256/2 (binary classification task, number of classes = 2)

**Table 3:** Comparison with other studies that used the DEAP dataset.

Author	Accuracies (%)
Chao et al. [43]	Val:68.28, Aro:66.73 (deep learning)
Pandey and Seeja [44]	Val:62.5, Aro:61.25 (deep learning)
Islam and Ahmad [45]	Val:81.51, Aro:79.42 (deep learning)
Alazrai et al. [46] Yang et al. [28]	Val:75.1, Aro:73.8 (traditional machine learn) Val:90.80, Aro:91.03 (deep learning)
Alhagry et al. [23] Liu et al. [47] Cui et al. [48] Vijiayakumar et al. [49] Li et al. [50] Luo [51] Zhong and Jianhua [52] Menezes et al. [53] Zhang et al. [54] Kumar et al. [55] Atkinson and Campos [56] Lan et al. [57] Mohammadi et al. [58] Koelstra et al. [39] Tripathi et al. [21] Cheng et al. [59] Wen et al. [60] Wang et al. [61] Gupta et al. [62] Yin et al. [27] Salama et al. [24] Our method	Val:85.45, Aro:85.65 (deep learning) Val:85.2, Aro:80.5 (deep learning) Val:96.65, Aro:97.11 (deep learning) Val:70.41, Aro:73.75 (traditional machine learn) Val:95.70, Aro:95.69 (traditional machine learn) Val:78.17, Aro:73.79 (deep learning) Val:78.00, Aro:78.00 (deep learning) Val:88.00, Aro:69.00 (deep learning) Val:94.98, Aro:93.20 (deep learning) Val:61.17, Aro:64.84 (Non—machine learning) Val:73.06, Aro:73.14 (deep learning) Val:73.10, Aro:71.75 (deep learning) Val:86.75, Aro:84.05 (deep learning) Val:57.60, Aro:62.00 (traditional machine learn) Val:81.40, Aro:73.40 (deep learning) Val:97.69, Aro:97.53 (deep learning) Val:77.98, Aro:72.98 (deep learning) Val:72.10, Aro:73.10 (deep learning) Val:79.99, Aro:79.95 (traditional machine learn) Val:85.27, Aro:84.81 (deep learning) Val:88.49, Aro:87.44 (deep learning) Val:99.99, Aro:99.98

## Data Availability

Data we use in this article is a public data set names”DEAP”. It is available for download via this link: https://www.eecs.qmul.ac.uk/mmv/datasets/deap/ (accessed on 1 April 2022).
